# Goblet Cells and Mucin Related Gene Expression in Mice Infected with *Eimeria papillata*

**DOI:** 10.1155/2013/439865

**Published:** 2013-12-03

**Authors:** Mohamed A. Dkhil, Denis Delic, Saleh Al-Quraishy

**Affiliations:** ^1^Department of Zoology, College of Science, King Saud University, P.O. Box 2455, Riyadh 11451, Saudi Arabia; ^2^Department of Zoology and Entomology, Faculty of Science, Helwan University, Cairo, Egypt; ^3^Department of Molecular Parasitology, Heinrich Heine University, Duesseldorf, Germany

## Abstract

Coccidiosis causes considerable economic loss in the poultry industry. The current study aimed to investigate the response of goblet cells as well as the induced tissue damage during *Eimeria papillata *infection. Mice were infected with sporulated *E. papillata* oocytes. On day 5 postinfection, the fecal output was determined. Also, the jejunum was prepared for the histological, histochemical, and molecular studies. Our results revealed that the intestinal coccidian infection with *E. papillata* induced a marked goblet cell hypoplasia and depleted mucus secretion. Also, the infection was able to alter the jejunal architecture and increased the apoptotic cells inside the villi. In addition, the real-time PCR results indicated that the inflammatory cytokines: TNF-**α**, iNOS, IFN-**γ**, and IL-1**β**, were significantly upregulated. In contrast, the mRNA expression patterns of IL-6 in response to *E. papillata* infection did not differ significantly between control and infected mice. Moreover, the mRNA expression of TLR4 was significantly upregulated, whereas the expression of MUC2 is significantly downregulated upon infection. Further studies are required to understand the regulatory mechanisms of goblet cells related genes.

## 1. Introduction

The intestine plays an important role in the digestion and absorption of ingested food and the elimination of undigested food, microbes, and microbial products [1]. The mucus layer coating the gastrointestinal tract is the front line of innate host defense, largely because of the secretory products of intestinal goblet cells [2].

Intestinal goblet cells are highly polarized secretory cells that are present throughout the intestinal tract. These specialized epithelial cells are thought to play an important protective role in the intestine by synthesizing and secreting several mediators, including the mucin MUC2 [3].

Indeed, infection with *Eimeria parasites* has been associated with increase in the incidence of the pathological conditions in poultry [4]. These parasites cause various problems ranging from gastroenteritis, anorexia, abdominal distention, diarrhoea, emaciation, and so forth, all of which result in serious economic losses to the farmer as well as the nation in general [4]. Since coccidioses lead to a significant impact on the livestock industry, we characterized the goblet cells response in mice inoculated with *Eimeria papillata* that infects enterocytes of the jejunum and shares many biological characteristics with the important pathogen of chickens, *E. tenella* [5].

The present study aimed to investigate the goblet cell response and its correlated genes during the infection with *E. papillata* in mice.

## 2. Materials and Methods

### 2.1. Animals

Twenty adult male Swiss albino mice weighing 35–30 g and aged 9–12 weeks were obtained from the animal facilities of King Saud University, Riyadh, Saudi Arabia. The mice were bred under specified pathogen-free conditions and fed a standard diet and water *ad libitum*. The experiments were approved by state authorities and followed Saudi Arabian rules for animal protection.

### 2.2. Experimental Design

Two groups of mice, with 10 animals per group, were investigated. The first group was inoculated only with sterile saline and served as the control group. The second group was orally infected with 10^3^ sporulated oocysts of *E. papillata*.

### 2.3. Infection of Mice

A self-healing strain of *E. papillata* was kindly provided by Professor Mehlhorn (Heinrich Heine University, Duesseldorf, Germany). Infected mice were orally inoculated with 1000 sporulated oocysts of *E. papillata* suspended in 100 *μ*L sterile saline. Subsequently, fresh faecal pellets were collected every 24 h. The collected pellets from each mouse were weighed and the bedding was changed to eliminate reinfection. Oocyst output was measured as previously described [6]. Faecal pellets were suspended in 2.5% (wt/vol) potassium dichromate and diluted in saturated sodium chloride for oocyst flotation. Oocysts were counted in a McMaster chamber and expressed as number of oocysts per gram of wet faeces.

### 2.4. Histological Analysis

Pieces of jejunum were freshly prepared from mice on day 5 postinfection with *E. papillata*, fixed in 10% neutral buffered formalin, and then embedded in paraffin. Sections were cut and then stained with hematoxylin and eosin. According to Dommels et al. [7], tissue sections were scored for inflammatory lesions (infiltrations by mononuclear cells, neutrophils, eosinophils, and plasmacytes, for fibrin exudation and lymphangiectasis), for tissue destruction (enterocyte loss, ballooning degeneration, edema, and mucosal atrophy), and for tissue repair (hyperplasia, angiogenesis, granulomas, and fibrosis). A rating score between 0 (no change from normal tissue) and 3 (lesions involved most areas and all the layers of the intestinal section including mucosa, muscle, and omental fat) was given for each aspect of inflammatory lesions, tissue destruction, and tissue repair. The sum of inflammatory lesions, tissue destruction, and tissue repair scores was used to represent the total histological injury score (HIS) for each intestinal section. The sum of the inflammatory lesions was multiplied by 2 to give more weight to this value since the tissue changes were mainly characterized by inflammatory lesions [7].

### 2.5. TUNEL Apoptosis Detection

Jejunum was collected on day 5 postinfection and terminal deoxynucleotidyl transferase mediated dUTP nick end labeling (TUNEL) assay staining was performed using a TUNEL Apoptosis Detection Kit (GenScript, Piscataway, NJ, USA) by following the manufacturer's protocol. Briefly, sections of paraffin-embedded jejunum were deparaffinized, rehydrated in gradient ethanol, and then digested with proteinase K. Slides were mounted with 4′,6-diamidino-2-phenylindole (DAPI). Using this procedure, positive nuclei of apoptotic cells are stained dark brown. Sections were counterstained with hematoxyline. For each animal, the number of apoptotic cells in the jejunum was counted on at least ten well-orientated villous-crypt units (VCU). Results were expressed as the mean number of apoptotic cells per ten VCU.

### 2.6. The Number of Goblet Cells

Sections were stained with Alcian blue for determination of the goblet cells. For each animal, the number of goblet cells in the jejunum was counted on at least ten well-orientated villous-crypt units (VCU). Results were expressed as the mean number of goblet cells per ten VCU [8].

### 2.7. Quantitative Real-Time PCR

Pieces of jejunum were aseptically removed, rapidly frozen, and stored in liquid nitrogen until use. Total RNA was isolated using Trizol (Invitrogen). RNA samples were treated with DNase (Applied Biosystems, Darmstadt, Germany) for at least 1 h and were then converted into cDNA by following the manufacturer's protocol using the reverse transcription kit (Qiagen, Hilden, Germany). Quantitative real-time PCR (qRT-PCR) was performed using the ABI Prism 7500HT sequence detection system (Applied Biosystems, Darmstadt, Germany) with SYBR green PCR master mix from Qiagen (Hilden, Germany). We investigated the genes encoding the mRNAs for the following proteins: interleukin-1*β* (IL-1*β*), interleukin-6 (IL-6), tumor necrosis factor alpha (TNF-*α*), interferon-*γ* (IFN*γ*), toll-like receptor 4 (TLR4), inducible nitric oxide synthase (iNOS), and mucin 2 (MUC2). All primer assays used for qRT-PCR were obtained commercially from Qiagen. PCRs were performed as follows: 2 min at 50°C to activate uracil-*N*-glycosylase (UNG); 95°C for 10 min to deactivate UNG; and 40 cycles at 94°C for 15 s, 60°C for 35 s, and 72°C for 30 s. Reaction specificity was checked by performing dissociation curves after PCR. mRNA levels were normalized to 18S rRNA. The fold induction of mRNA expression upon infection with *E. papillata* was determined using the 2−ΔΔCT method [9].

### 2.8. Statistical Analysis

A two-tailed Student *t*-test was used for statistical analysis.

## 3. Results

The course of *E. papillata* infections in mice was recently characterized in detail [10, 11]. The number of excreted oocysts varied among the individual mice between 200,000 and 260,000 per gram feces (mean value, 235,000 ± 24,000).

**Figure 1:**
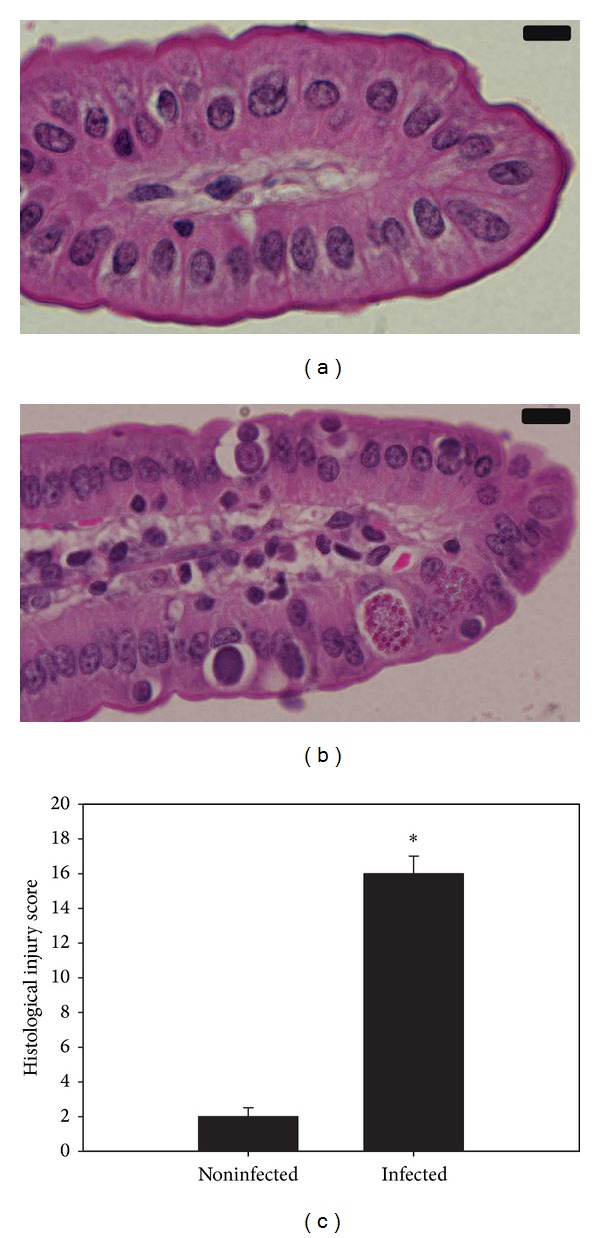
*E. papillata*-induced jejunum injury on day 5. (a) Noninfected jejunum with normal architecture of the absorptive epithelium and lamina propria. (b) Infected jejunum with some pathological changes in lamina propria and absorptive epithelia. Developmental stages appearing in the absorptive epithelia. Sections are stained with hematoxylin and eosin. Bar = 25 *μ*m. (c) Total histological injury score in jejunum of mice infected with *E. papillata* on day 5 p.i. Values are means ± SD.  *Significant at *P* ≤ 0.05. *n* = 10.

Light microscopical inspection of hematoxylin-and-eosin-stained sections revealed that the epithelial cells of the jejunum were infected by *E. papillata*. Concomitantly, there occurred some histological changes which were semiquantified by applying the scoring according to Dommels et al. [7]. Histological analysis revealed that mice infected with sporulated oocysts of *E. papillata* suffered a moderate inflammatory injury in jejunum (Figure 1).

**Figure 2:**
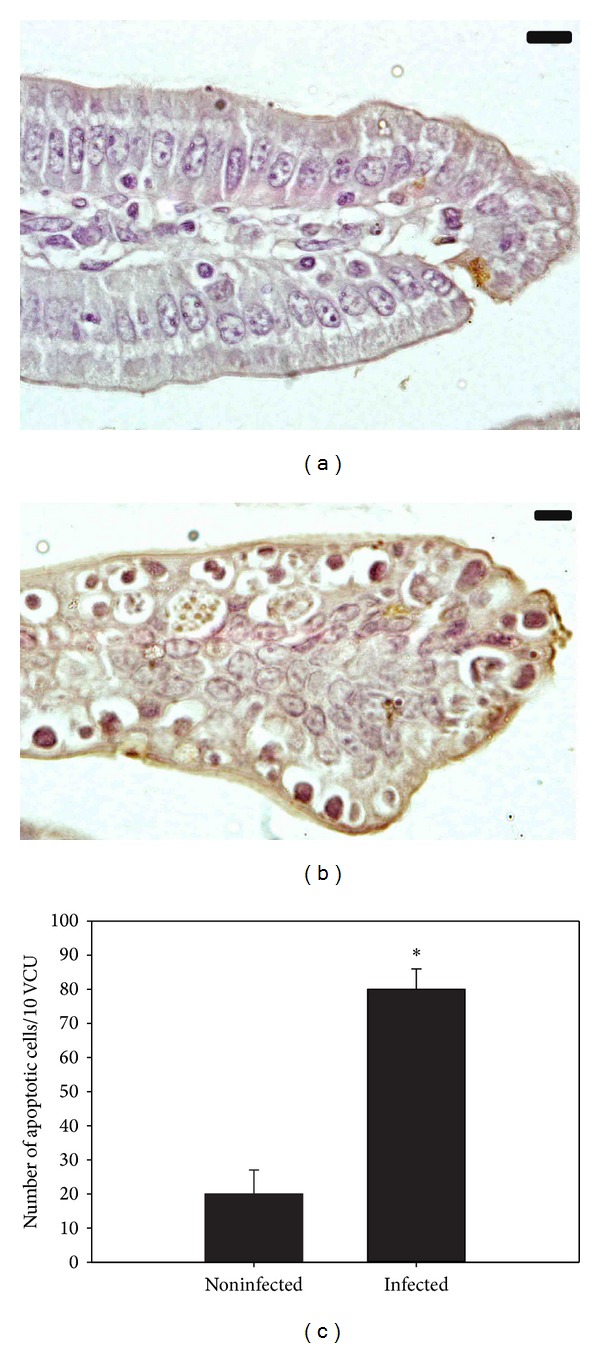
Changes in apoptotic cells in mouse jejunum infected with *E. papillata*. (a) Noninfected jejunum (b) Infected jejunum with increased number of TUNEL-positive cells in lamina propria. Bar = 25 *μ*m. (c) Infection-induced changes in apoptotic cells number in jejunal crypts. Values are means ± SD.  *Significant at *P* ≤ 0.05. *n* = 10.

**Figure 3:**
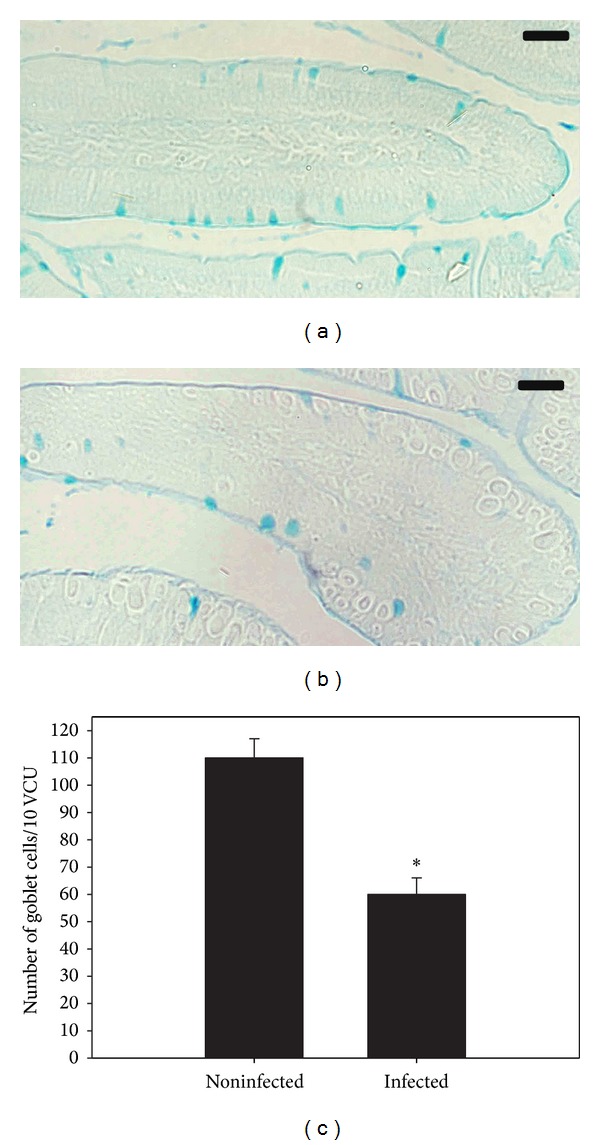
Changes in goblet cell numbers in mouse jejunum infected with *E. papillata*. (a) Noninfected jejunum with more goblet cells. (b) Non-infected treated mouse jejunum. Sections are stained with Alcian blue. Bar = 25 *μ*m. (c) Number of goblet cells in jejunum of mice at day 5 p.i. Values are means ± SD.  *Significant at *P* ≤ 0.05. *n* = 10.

Infection of mice with *E. papillata* induced a significant increase in the number of TUNEL-positive cells of the jejunal crypts compared to the noninfected controls (Figure 2). In addition, there was a significant reduction of the goblet cell numbers seen at the site of the *E. papillata* infection in the jejunum (Figure 3).

**Figure 4:**
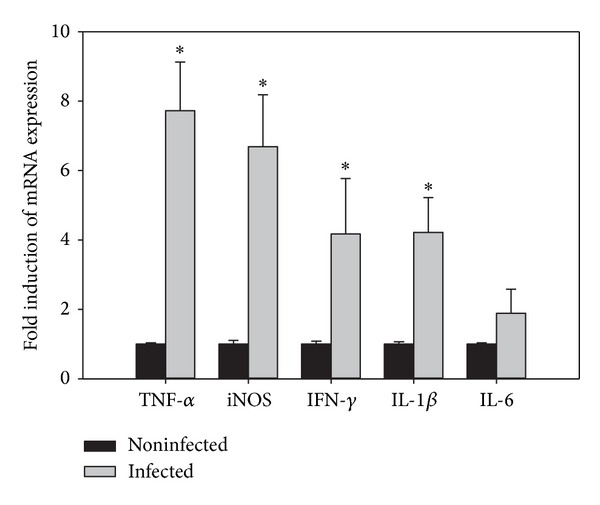
Quantitative RT-PCR analysis of TNF-*α*, iNOS, IFN-*γ*, IL-1*β* and IL-6 in the jejunum. Expression was analyzed in noninfected and infected mice on day 5 p.i., normalized to 18S rRNA signals, and relative expression is given as fold increase compared to the non-infected control mice. Values are means ± SD.  *Significant change at *P* < 0.01 with respect to non-infected mice.

**Figure 5:**
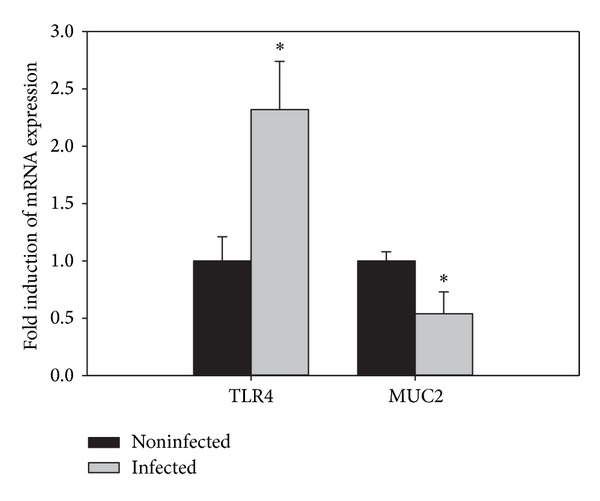
Quantitative RT-PCR analysis of TLR4 and MUC2 in the jejunum. Expression was analyzed in non-infected and infected mice on day 5 p.i., normalized to 18S rRNA signals, and relative expression is given as fold increase compared to the non-infected control mice. Values are means ± SD.  *Significant change at *P* < 0.01 with respect to noninfected mice.

Quantitative real-time PCR was used to detect changes in the mRNA levels of inflammatory and goblet cell associated genes in the intestine. Upon infection with *E. papillata*, there was a significant increase in the mRNA expression of TNF-*α*, iNOS, IFN-*γ*, and IL-1*β*. In contrast, the mRNA expression patterns of IL-6 in response to *E. papillata* infection did not differ significantly between control and infected mice (Figure 4). The mRNA expression of TLR4 is significantly upregulated, whereas the expression of MUC2 is significantly downregulated upon infection (Figure 5).

## 4. Discussion

Intestinal infection with *E. papillata* can induce substantial pathological changes in the epithelial compartment [11], as leads to a dramatic reduction in the number of goblet cells [11]. The mucus released by goblet cells can function as a defensive barrier [12, 13]. Our results demonstrated that goblet cells were most evident in the infected crypt and much less so in the neighboring and other uninfected areas of the intestine. In addition, the parasites were mostly discovered at the bottom of the intracrypt epithelium.

In accordance with previous studies, the intracellular development of these parasites in the jejunum is rapid thus resulting in fecal output of *Eimeria* oocysts [14]. Epithelial host cells finally disrupt upon discharging the oocysts, and tissue inflammation is therefore expected to occur during *E. papillata* infections. Also, our data have revealed that the inflammatory process occurring in mice gut is strong and is exacerbated by protozoal invasion. This is not only recorded by histology but also evidenced by the upregulation of the inflammatory cytokines: TNF-*α*, iNOS, IFN-*γ*, and IL-1*β*.

Only IFN-*α*, which is known to activate intracellular cytotoxicity [15], is strongly increased during the infection suggesting a specific role of IFN-*α* in *E. papillata* infection. Indeed, previous findings have also shown an increased production of IFN-*α*, mainly by NK-cells, during primary infections with *E. papillata* [14]. Also, a strong IFN-*α* response has been described to occur in the intestine upon infection with *E. maxima* [16], *E. bovis*, and *E. alabamensis* [17]. It has been suggested that IFN-*α* limits the output of oocysts during primary infection with *E. papillata* [14]. Also, the changes in goblet cell numbers may affect the susceptibility of the parasite-infected host to limit the capacity of opportunistic pathogen from increasing or penetrating the local epithelium [18].

Apoptosis is normal part of development and tissue homeostasis [19]. Apoptotic cells are present in the intestinal crypts and regulate the total amount of progenitor stem cells [20]. The great increase in the amount of apoptosis within *E. papillata* infected villi may be due to the complex host-parasite interaction. Moreover, apoptosis is an important regulator of the host's response during various intracellular protozoan infections and helps eliminate damaged or infected cells [21].

Our results of qRT-PCR revealed that the expression of MUC-2 is significantly reduced, whereas the expression of MUC-4 is not changed. MUC-2 is the first line of innate host defense in preventing pathogen-induced epithelial injury. In the absence of MUC-2, mice are more susceptible to *Entamoeba histolytica*-induced secretory and proinflammatory responses [22]. Moreover, MUC2^−/−^ mice spontaneously develop colitis, indicating that MUC2 is essential for the protection of the colon [23]. The inhibition of the goblet cell marker MUC-2 correlates with an activation of the toll-like receptor 4 (TLR4) (Figure 5). Recently, it was shown that intestinal epithelial TLR4 regulates goblet cell development [24]. In our study, *E. papillata* induced an upregulation of TLR4 and a downregulation of MUC2 which is consistent with the previous observation. Moreover, *E. papillata* induces an upregulation of the four miRNA species: miR-1959, MCMV-miR-M23-1-5P, miR-203, and miR-21 [25]. Unfortunately, *in silico* analysis using the miRWalk database [26] revealed that none of our deregulated genes are a validated target of the described miRNAs.

Collectively, our data suggest that infections of mice with *E. papillata* not only induce pathogenesis in the jejunum, the final target site of *E. papillata*, but also induced an upregulation of TLR4 and a downregulation of MUC2. Further studies are required to know the mechanism of goblet cells regulated genes during infection with *E. papillata*.
